# Supplementary material for: The Synergistic Effects of Curcumin and Chemotherapeutic Drugs in Inhibiting Metastatic, Invasive and Proliferative Pathways
Source: Plants (Basel). 2022 Aug 17;11(16):2137. doi: 10.3390/plants11162137 (PMC9414747; doi:10.3390/plants11162137)
Supplement: Supplementary file 1 [file plants-11-02137-s001.zip › plants-1844555-supplementary.pdf]

Table S1. Table summarizing the combination therapy in the past five years of curcumin and chemotherapeutic agents (radiation, 5-fluorouracil, doxorubicin, paclitaxel, metformin, docetaxel, berberine, gemcitabine, carboplatin, resveratrol, DCA, luteolin, aprepitant, WFB, thymoquinone and DIM) in metastatic, invasive, and proliferative cancer therapy.

Reference	Cancer Type (cell lines)	Type of Intervention	Methods	Molecular Outcome	Study Conclusion
**[36]**	Glioblastoma (LN18)	Pharmacological: Curcumin + Light Radiation	In vitro: Cell viability assay + Transwell chamber assay	• Reduced proliferation of LN18 cells<br>• Inhibition of invasion upon exposure to high and low LET<br>• Higher sensitivity of LN18 to high or neutron LET	The combination of curcumin with either neutron- or γ-LET inhibits invasion of LN18 with a higher sensitivity if glioblastoma cells to neutron high- radiations.
**[35]**	Bladder Cancer (RT112, UMUC-3, TCCSUP)	Pharmacological: Curcumin + Light Radiation	In vitro: Integrin surface expression + Western Blot+ Blocking Studies	• Exerted synergistic effect.<br>• Suppression of chemotaxis in both cell lines.<br>• Reduced expression of integrin subtypes (α2, α3, α5, α6, β1, and β3) in UMUC-3.<br>• Reduced expression of integrin subtypes α3, α6, and β1 in RT112.<br>• Reduced expression of integrin subtypes (α2, α3, α5, β1, and β3) in TCCSUP.<br>• Suppression of pFAK by CUR+ Light.<br>• Inhibition of the cancerous cells to bind to fibronectin and collagen.	The combination of curcumin and light radiation had a vital role in the repression of cancerous cells to adhere and migrate to other cells by inhibiting the expression of multiple integrins in different cell lines in bladder cancer.
**[33]**	Renal Cell Carcinoma (A498, Caki1, KTCTL-26)	Pharmacological: Curcumin + Light Radiation	In vitro: ELISA+ Western Blot	• Exerted synergistic effect.<br>• Blocked adhesion of cells to HUVECs.<br>• Downregulation of chemotaxis in all three cell lines.<br>• Increased expression of α3, β1, and β3 and decreased expression of α2, α4, α6 in A 498 cells.<br>• Strong expression of α3, β1, α5, β3 while weak expression of α1 and α4 were detected in Caki1 cell lines.	Curcumin and light radiation obstructed the different cell lines of RCC to adhere to HUVEC while also permitting the downregulation of the cell lines to attach to the cell matrix thus inhibiting migratory and invasive properties.

				• Strong expression of α3, β1 while moderate expression of α5, β3, α1, α2, α6, and β4 were detected in KTCTL-26 cell lines.	
**[32]**	Lung Cancer (A549)	Pharmacological: Curcumin + Light Radiation	In vitro: Transwell invasion and Wound healing Assays + Western Blot + ELISA	• Exerted synergistic effect.<br>• CUR reversed resistance of the cells to radiation and increased their sensitivity which lead to a decrease in proliferation.<br>• Decrease in E-cadherin, vimentin and<br>• Slug expression levels.<br>• Upregulation of Keratins.<br>• Inhibition of MMP9 expression.	Curcumin enhanced the sensitivity of the cell lines to light radiation which in turn enhanced the anti-metastatic and anti-invasive properties of the cell lines by the inhibition and downregulation of MMP9, E-cadherins, and SLUG expression levels.
**[34]**	Prostate Cancer (DU145, PC3)	Pharmacological: Curcumin + Light Radiation	In vitro: ELISA + Western Blot	• Exerted synergistic effect<br>• Reduced proliferation in both cell lines.<br>• Suppression of motility of both cell lines.<br>• Downregulation of CD44 cell receptor adhesion.<br>• Downregulation of α5, α6, αV on both cell lines.<br>• Reduced expression of β3 and β4 on DU145 and PC3 respectively.<br>• Loss of ILK and FAK upon PC3 death by CUR+ radiation.	Curcumin in combination with light radiation decreased cell proliferation as well as reduced cell adhesion and invasion by the modification of cell specific integrins.
**[42]**	Gastric cancer (AGS)	Pharmacological: Curcumin + Doxorubicin	In vitro: MTT cell viability assay, wound-healing assay and transwell migration assay	• Decrease in AGS cell viability<br>• Dose-dependent inhibition of cell invasion and migration	Curcumin and doxorubicin exhibit together anti-invasion and anti-migration activity on AGS cells in vitro
**[43]**	Breast Cancer (MDA-MB-231)	Curcumin + Doxorubicin	In vitro: Cytotoxicity assay, transwell	• Exerted synergistic effect	Curcumin + Doxorubicin inhibits invasion. Migration and metastasis of breast cancer cells

			invasion assay, transendothelial migration assay, migration assay<br>In vivo: Pulmonary metastasis analysis	• Dose-dependent anti-proliferative effect of CUR+DOX on TNBC<br>• Inhibition of tumor invasion<br>• Inhibition of transendothelial migration hence suppressing tumor metastasis<br>• Suppression of pulmonary metastasis	
**[44]**	Colorectal cancer (SW480, HT-29)	Pharmacological: Curcumin + 5-Fluoruracil	In vitro: CCK-8 Assay+ Western Blot	• Exerted synergistic effect.<br>• Downregulation of NNMT by p-STAT3 depression.<br>• CUR increased sensitivity of NNMT to 5FU.	CUR+5-FU inhibit CRC proliferation (in metastatic organs) and diminish resistance to 5-FU individually.
**[45]**	Hepatocellular Carcinoma (SMMC-7721, Bel-7402, HepG-2, MHCC97H L02)	Pharmacological: Curcumin+ 5-Fluorouracil<br><br>Non-pharmacological: Mice	In vivo: Intraperitoneal injections into mice.<br><br>In vitro: Cell inhibition assay+ Western Blot	• Exerted Synergistic effect.<br>• Decreased expression of NF-κB protein in the nucleus.<br>• Increased expression of NF-κB protein in cytoplasm.<br>• Downregulation of COX-2 protein by CUR+5-FU.<br>• Growth inhibition of tumor in vivo.<br>•	CUR+5FU reduce proliferation of cells by preventing the movement of NF-κB from the cytoplasm to the nucleus and reducing COX-2 expression.
**[47]**	Colorectal cancer (HCT-116)	Pharmacological: Curcumin + 5Fluorouracil	In vitro: MTT Assay+ Western Blot	• Exerted synergistic effect.<br>• Inhibition of proliferation through CUR+5FU.<br>• Inhibition of EMT by WNT downregulation and NKD2 upregulation.<br>• Reduced β-catenin, E-cadherin, TCF4and Axin expression through.<br>• Increased expression of TET1, NKD2 and Vimentin.<br>• Expression of Pax-6	CUR+5FU inhibited the Wnt signal pathway and EMT process through Pax-6 expression, thus inhibiting cell migration and invasion.
**[50]**	Human ovarian cancer (SKOV3 and SKOV3-TR30)	Curcumin+ Paclitaxel	In Vitro: Preparation (PEI-SA)HA Micelles+ Cytotoxicity Assay+	• Exerted synergistic effect.<br>• (PEI-SA)HA/PC inhibited the cell invasiveness of SKOV3 and SKOV3-TR30 significantly.	Curcumin+ Paclitaxel inhibited metastasis in a dose-dependent manner

			Migration Experiment	• Metastasis was inhibited in a dose-dependent manner.	
**[52]**	Breast Cancer (4T1 and MDA-MB-231)	Curcumin+ Paclitaxel	Formation of gold nanoparticles + apoptotic assay+ DCFDA assay+ Transwell migration assay + Flow cytometry	• Decreased expression of VEGF, Cyclin D, and STAT3 genes<br>• Enhanced caspase 9 gene expression<br>• Retardation in MDA-MB 231 cell migration	Inhibition of metastasis
**[55]**	Breast Cancer (TNBC)	Quercetin+ Berberine+ Curcumin	In vitro: Migration assay+ Western Blotting + Immunofluorescence+ MTT assay	• Inhibition of EMT proteins in TNBC cells<br>• Significant decrease in cell migration<br>• Decreased cell growth in 3D culture<br>• Significant inhibition of breast cancer cells<br>• Decreased expression of β-catenin, CD44, and MMP-9 proteins, compared to the controls.<br>• Inhibition of the expression of EMT markers	Combination treatment significantly inhibits the migration of TNBC cells.
**[56]**	Breast Cancer (MCF7)	Pharmacological: Berberine + 5-Fu + Curcumin	In vitro: Transwell chamber assay	• Stronger effect when berberine and 5-Fu were combined with Curcumin<br>• Inhibition of MCF-7 cells invasion	Combination treatment significantly inhibits the migration of MCF-7 cells
**[57]**	Pancreatic cancer (PANC-1)	Pharmacological: gemcitabine and docetaxel + curcumin	In vitro: Matrigel invasion assay	• Gemcitabine+ curcumin: ↓ of invaded PC cells.<br>• ↓ in MMP2 and MMP9 expression.<br>• ↑ in TIMP1 expression.<br>• Docetaxel + curcumin: little inhibition of PANC-1 invasive cells.<br>• ↓ in MMP2 and MMP9 expression.<br>• ↑ in TIMP1 expression.	Both drugs in combination with curcumin have synergistic effects: target MMPs which are key drivers in cancer progression (metastasis) and promote TIMP1 expression, a natural inhibitor of MMPs
**[60]**	Glioma (U87 glioma cells and bEND.3	curcumin and docetaxel co-loaded poly lactide-*co*-	In vitro MTT assay	• DTX+ CCM loaded PLGA NP lead to a drop in IC50 IN U87 cells	A lower IC50 reflects a more potent drug leading to an inhibition in invasion and therefore metastasis

	endothelial cells)	glycolide (PLGA) nanoparticles			
**[59]**	Pancreatic cancer (LNCaP)	Intravenous injection: GE11-DTX-CUR NPs	In vitro Cytotoxicity evaluation (MTT assay) and in vivo studies using mice	• GE11-DTX-CUR NPs showed maximum decrease of IC_{50} values thus inhibiting cell proliferation<br>• and were effective in slowing tumor progression (High TIR)	CUR inhibit the PI3K/AKT pathway and induce low levels of ER-stress and along DTX assembled as GE11-DTX-CUR NPs constitutes a synergistic antitumor treatment.
**[58]**	Esophageal squamous cell carcinoma (ESCC, KYSE150 and KYSE510)	Pharmacological: docetaxel+ curcumin (DC)	In vitro Invasion assay Scratch wound healing assays	• DTX+ CUR: weaken healing ability of esophageal cancer cells and inhibit their invasion.	The findings of scratch healing experiments and Transwell assays showed that DC significantly impaired the scratch-healing ability of esophageal cancer cells and enhanced the ability of DTX to inhibit invasion and metastasis of esophageal cancer cells.
**[61]**	Hepatocellular carcinoma (HepG2)	Pharmacological: metformin+ curcumin	In vitro: Matrigel invasion assay Wound scratch assay Gelatin zymography protease assay	• ↑ in inhibition rate of invasion for HepG2 cells and PLC/PRF/5 cells using 5mM metformin and 2.5/5 μM curcumin.<br>• Inhibition of active MMP9 and MMP2 in HepG2 cells and xenografts.<br>• ↑ expression of PTEN and p53.<br><br>• Inhibition of NF-kB.	A combined treatment of metformin with curcumin inhibits invasion of HepG2 or PLC/PRF/5 cells and downregulate the expression of MMP9 and MMP2 in HepG2 cells affecting metastasis.<br><br>Synergistically inhibits PTEN/PI3K/Akt/mTOR signaling and also suppresses NF-κB nuclear translocation
**[62]**	Gastric adenocarcinoma	Pharmacological: metformin+ curcumin	In vitro: Cell invasion assay	• Metformin +curcumin<br>• ↓ in invasion cell number<br>• significant inhibitory effects of single and combinatorial treatments on cell invasion rates.	Significant inhibitory effect of metformin and curcumin combinatorial treatment on cell invasion rates inhibiting metastatic potential of AGS cells
**[64]**	Pancreatic cancer	Pharmacological: gemcitabine + curcumin (Meriva)	Phase II clinical trial	• High baseline levels of IL-6 and sCD40L in metastasized cancer<br>• In patients responding to the therapy no significant variations of biomarkers were found between baseline levels	Gemcitabine in combination with Meriva resulted in the absence of neurotoxicity and lower hematological toxicity and contribute to preserve quality of life

**[65]**	Pancreatic cancer	Pharmacological: gemcitabine + curcumin	In vitro Migration assay<br>In vivo orthotopic mouse model<br><br>Bioluminescence imaging (BLI) method to detect tumor growth and metastasis	• SP-CUR+ GEM:<br>• ↑ of E-cadherin which inhibits metastasis<br>• None of the mice treated with SP-CUR + GEM were recorded for distant metastasis<br>• SP-CUR inhibits aberrant SHH expression, CXCL12-induced CXCR4 expression was suppressed	Co-treatment of SP-CUR and GEM in a pancreatic orthotopic mice restricts tumor growth, metastasis and improves survival by targeting the SHH pathway.
**[66]**	Lung cancer	Pharmacological: gemcitabine + curcumin	In vitro: Transwell migration assay and wound healing assay	• Improved sensitivity of A549 gemcitabine-resistant cells<br>• Inhibition of invasion of A549 cells<br>• Inhibition of migration of A549 cells<br>• ↓ in the expression of pro-invasion markers: MMP9, Vimentin, N-cadherin<br>• ↑ of E-cadherin which inhibits metastasis	Combination of gemcitabine with curcumin inhibits invasion and migration of A549 lung cancer cells
**[67]**	Lung cancer	Pharmacological: carboplatin+ curcumin	In vitro Scratch wound-healing motility assay<br>Invasion assay<br>Matrix metalloproteinase activity assay	• Inhibition of cell invasion and migration<br>• 100 µM carboplatin + curcumin: inhibition of MMP-2 and MMP-9 activity<br>• ↓in MMP-9 mRNA expression	Combination of curcumin and carboplatin has a synergistic antimetastatic effect in A549 cells through inhibition of MMP-2 and MMP-9.
**[68]**	Triple negative breast cancer	Pharmacological: carboplatin+ curcumin	In vitro Colony formation assay<br>MTT assay<br>Western blot<br>Flow cytometry	• Inhibit proliferation and reduce colony formation<br><br>• ↓ RAD51 expression (resistance) and ↑ γH2AX expression (DNA damage marker) and ROS levels -> NAC used to counteract	Curcumin attenuated the expression of the DNA repair protein RAD51 by increasing ROS levels to enhance the response to carboplatin. Carboplatin used with curcumin can slow metastasis and increase survival rate by minimizing the side effects.

**[69]**	Colorectal cancer cells	Pharmacological: Curcumin + resveratrol	In vitro: Staining for mitochondrial membrane potential and plasma membrane integrity Molecular docking	• Promotes apoptosis by inducing mitochondrial instability in both cell lines<br>• Promotes apoptosis via caspase dependent pathway<br>• P53-independent cell death activity of curcumin + resveratrol<br>• G0/G1 cell cycle arrest upon treatment with both drugs<br>• Alteration in MMP7 protein upon combination therapy	Combination of curcumin and resveratrol has a synergistic antiproliferative, antimetastatic and pro-apoptotic effects in colorectal cancer cells
**[70]**	Breast Cancer cells	Curcumin + dichloroacetate	In vitro MTT assay, Colony formation assay, molecular docking	• Antiproliferative activity of combination therapy on cancer cells<br>• Curcumin and DCA inhibits colony formation in both cell lines	Curcumin and Dichloroacetate inhibit breast cancer cells survival, proliferation and metastasis
**[71]**	Colon cancer cells	Curcumin + Luteolin	In vitro: cell proliferation assay, wound-healing assay, Western blot	• Synergistic effect of curcumin and luteolin on colon cancer cells<br>• Anti-proliferative effect on both cell lines<br>• Inhibition of tumor metastasis revealed by wound-healing assay<br>• Suppression of Notch-1 and TGF-β	Curcumin and Luteolin synergistically inhibit the proliferation as well as invasion and metastasis of colon cancer cells via the suppression of the Notch-1 and TGF-β signaling pathway
**[73]**	Hepatocellular carcinoma	Curcumin + Aprepitant	In vitro: Cytotoxicity assay, cell migration assay<br>In vivo: Lung metastasis	• Efficient delivery of both drugs via the liposomes conjugates<br>• Anti-proliferative effect<br>• Inhibition of metastasis revealed by wound healing assay<br>• Reduced ECM deposition via the inhibition of collagen IV production<br>• Inhibition of lung metastasis in when mice were treated with both drugs as compared to control groups	Co-delivery of curcumin and aprepitant via modified liposome conjugates, effective in suppressing tumor cell proliferation, invasion and metastasis as well as inhibiting lung metastasis in vivo
**[74]**	Lung and Liver cancer cells	Curcumin + Thymoquinone + 3,3′-	In vitro: cell proliferation assay,	• Antiproliferative effect of combinatorial treatment on lung and liver cancer cells	Combination of curcumin with thymoquinone and DIM exhibits anti-proliferative as

		diindolymethane (DIM)	migration assay, colony formation assay and Western blot analysis	• Inhibition of A549 and HepG2 cell migration<br>• Decreased expression of PI3K and AKT proteins level	well as anti-metastatic activity on lung and liver cancer cells
**[75]**	Nasopharyngeal carcinoma cells (NCC)	Curcumin + Wikstroflavone B (WFB)	In vitro: Cytotoxicity assay, colony formation assay, wound healing assay and transwell migration assay	• Anti-proliferative activity on NCC<br>• Dysregulation of cyclin D1, surviving, p53 and p21<br>• Anti-metastatic activity on NCC<br>• Downregulation of MMP-2 and MMP-9<br>• Inhibition of the FAK/STAT3 signaling pathway	Curcumin when combined with WFB inhibits nasopharyngeal carcinoma cell proliferation, invasion and metastasis via the suppression of the FAK/STAT3 signaling pathway
